# SRT2379, a small-molecule SIRT1 activator, fails to reduce cytokine release in a human endotoxemia model

**DOI:** 10.1186/cc12909

**Published:** 2013-11-05

**Authors:** Maryse A Wiewel, Anne Jan van der Meer, Jonathan Haddad, Eric W Jacobson, George P Vlasuk, Tom van der Poll

**Affiliations:** 1 Center for Experimental and Molecular Medicine, Academic Medical Center, University of Amsterdam, the Netherlands; 2 Sirtris, A GSK Company, Cambridge, MA, USA

## Background

SRT2379 is a selective small-molecule activator of the NAD^+^-dependent deacetylase, Sirtuin 1 (SIRT1), which has broad anti-inflammatory effects in cell cultures and rodents. The aim of the current study (EUDRACT # 2011-002266-20) was to determine the effect of SRT2379 on the inflammatory responses in normal healthy male subjects after exposure to LPS.

## Materials and methods

This single-blind, placebo-controlled study consisted of four treatment arms (*n *= 8 per arm): (1) oral SRT2379 50 mg; (2) oral SRT2379 250 mg; (3) oral SRT2379 1000 mg; and (4) placebo. All subjects received a single dose of study drug on day 1 followed by intravenous LPS 4 hours later. Laboratory parameters of inflammation along with assessment of clinical signs, safety assessments, and pharmacokinetic measurements were recorded at baseline and after LPS administration.

## Results

SRT2379 was well tolerated. Adverse events were similar across all treatment groups and were predominantly as expected with LPS administration. Pharmacokinetic exposures increased in a dose-dependent manner. SRT2379 did not significantly impact cytokine release as compared with placebo: TNFα (183.52, 177.57, 123.84 vs. 195.30 pg/ml for groups 1, 2, 3 vs. group 4, respectively, *P *> 0.05), IL-6 (195.25, 237.51, 180.26 vs. 250.08 pg/ml, respectively, *P *> 0.05), IL-17 (3.88, 2.59, 6.42 vs. 8.09 pg/ml, respectively, *P *> 0.05), IL-8 (126.11, 105.25, 110.56 vs. 108.77 pg/ml, respectively, *P *> 0.05), and IL-10 (12.61, 13.03, 40.40 vs. 11.90 pg/ml, respectively, *P *> 0.05). SRT2379 also had no impact on vital signs, leukocyte counts, or coagulation activation markers compared with placebo.

## Conclusions

Although SRT2379 suppresses inflammatory markers in preclinical experiments, we were unable to demonstrate a similar impact in this human model of endotoxemia. This may be due to potency or exposure issues, with the compound. SRT2379 terminated for further clinical development. More promising candidates are being identified for future clinical exploration.

